# Cannabidiol in Periodontal Therapy—Is There Hope or Just a Bias? A Systematic Review

**DOI:** 10.3390/biomedicines14051163

**Published:** 2026-05-20

**Authors:** Ruxandra Ștefănescu, Amelia Tero-Vescan, Camil-Eugen Vari, Dragoș Sita, Bianca-Eugenia Ősz

**Affiliations:** 1 Department of Pharmacognosy and Phytotherapy, Faculty of Pharmacy, George Emil Palade University of Medicine, Pharmacy, Science, and Technology of Targu Mures, 540142 Targu Mures, Romania; ruxandra.stefanescu@umfst.ro; 2 Department of Biochemistry, Faculty of Medicine in English, George Emil Palade University of Medicine, Pharmacy, Science, and Technology of Targu Mures, 540142 Targu Mures, Romania; 3 Department of Pharmacology and Clinical Pharmacy, Faculty of Pharmacy, George Emil Palade University of Medicine, Pharmacy, Science, and Technology of Targu Mures, 540142 Targu Mures, Romania; camil.vari@umfst.ro (C.-E.V.); bianca.osz@umfst.ro (B.-E.Ő.); 4 Department of Odontology and Oral Pathology, Faculty of Dental Medicine, George Emil Palade University of Medicine, Pharmacy, Science, and Technology of Targu Mures, 540142 Targu Mures, Romania; dragos.sita@umfst.ro

**Keywords:** cannabidiol, periodontitis, periodontal inflammation, biofilm, antimicrobial activity, host modulation

## Abstract

**Background:** Periodontitis is a chronic inflammatory disease characterized by dysbiotic biofilm formation, progressive destruction of periodontal tissues, and alveolar bone resorption. Conventional periodontal therapy primarily focuses on mechanical biofilm removal; however, adjunctive therapeutic approaches targeting host inflammatory responses and microbial activity have gained increasing attention. Cannabidiol (CBD), a non-psychoactive phytocannabinoid derived from *Cannabis sativa*, has demonstrated anti-inflammatory, antimicrobial, and immunomodulatory properties that may be relevant in periodontal disease management. **Objective:** This systematic review aimed to evaluate the available evidence regarding the potential role of CBD in modulating periodontal inflammation, microbial biofilms, and bone resorption processes. **Methods:** A systematic literature search was conducted in Web of Science, Cochrane, PubMed, Scopus, and Google Scholar. The review was conducted in accordance with PRISMA guidelines. Studies investigating the effects of CBD on periodontal inflammation, oral biofilms, or bone remodeling were included. Both preclinical (in vitro and animal) and clinical studies were considered. **Results:** Evidence from experimental studies consistently demonstrated that CBD modulates inflammatory signaling pathways, including inhibition of the TLR4/NF-κB pathway and a reduction in pro-inflammatory cytokine expression, but some results are contradictory. Animal studies reported reduced alveolar bone loss and decreased osteoclast activity following CBD administration. Several studies also demonstrated antimicrobial and antibiofilm effects of CBD against oral microorganisms. **Conclusions:** While preclinical evidence is promising, the current body of clinical data remains limited. Further well-designed randomized clinical trials are required to determine the efficacy, type of formulation, optimal dosing, and long-term safety of CBD as an adjunctive therapy in periodontal treatment.

## 1. Introduction

Periodontitis is a chronic inflammatory disease affecting the supporting structures of the teeth, including the periodontal ligament, gingiva, and alveolar bone. The disease is initiated by the accumulation of dysbiotic subgingival biofilm and is characterized by a destructive host immune response that leads to progressive connective tissue breakdown and alveolar bone resorption. If untreated, periodontitis may ultimately result in tooth mobility and tooth loss [1,2,3].

The pathogenesis of periodontitis involves a complex interaction between microbial pathogens and host immune responses. Key periodontal pathogens such as *Porphyromonas gingivalis*, *Treponema denticola*, and *Aggregatibacter actinomycetemcomitans* contribute to the establishment of a dysbiotic microbial environment that stimulates persistent inflammation [4,5,6]. In response to bacterial challenge, host immune cells release pro-inflammatory mediators, including cytokines such as interleukin-1β (IL-1β), tumor necrosis factor-α (TNF-α), and interleukin-6 (IL-6). These mediators activate signaling pathways such as NF-κB, leading to increased expression of matrix metalloproteinases and osteoclast differentiation factors that ultimately drive periodontal tissue destruction [7,8,9]. Given the central role of inflammation and microbial biofilms in periodontal disease progression, therapeutic strategies targeting both microbial load and host inflammatory responses have gained increasing interest in recent years [10,11].

The current standard of care for periodontitis primarily relies on mechanical debridement, including scaling and root planning, to remove microbial biofilms and calculus deposits. Adjunctive antimicrobial agents, such as Chlorhexidine, are often used to control bacterial growth and reduce plaque accumulation [1,12].

Although these approaches are generally effective, several limitations remain. Mechanical therapy alone may not fully eliminate pathogenic biofilms in deep periodontal pockets, and long-term use of antiseptic agents may lead to adverse effects, including tooth staining, taste disturbances, and mucosal irritation. Furthermore, conventional antimicrobial approaches do not directly address the host inflammatory response that contributes to tissue destruction [13,14].

For these reasons, there has been increasing interest in host-modulatory therapies that aim to regulate inflammatory pathways involved in periodontal disease progression.

**Figure 1:**
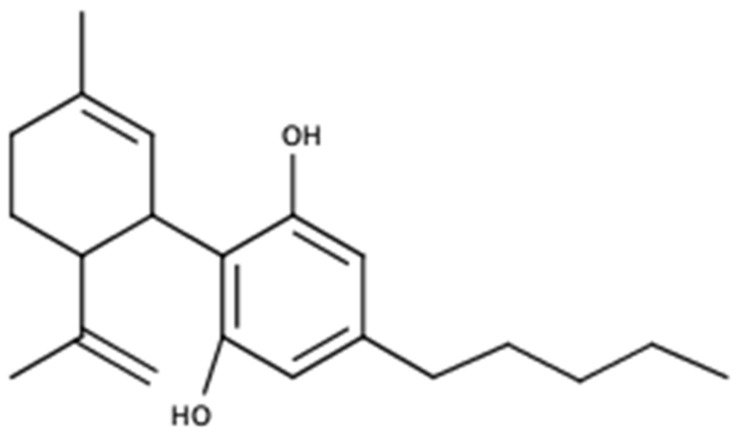
The structure of cannabidiol (CBD).

Cannabidiol (CBD) is one of the main non-psychoactive phytocannabinoids found in *Cannabis* sp. The structure of this terpenophenol (Figure 1) was elucidated by Mechoulam and Shvo in 1963, and since then has been the topic of over 2000 studies [15]. The biological effects of cannabinoids are largely mediated through the endocannabinoid system, which includes cannabinoid receptors (CB1 and CB2), endogenous ligands, and enzymes involved in cannabinoid metabolism [16,17]. Cannabinoid receptors are expressed in several cell types relevant to periodontal disease, including immune cells, fibroblasts, and osteoclasts. Activation of these pathways has been associated with modulation of inflammatory responses, oxidative stress, and bone metabolism [18,19,20]. However, the multitude of products with CBD that appeared in recent years is leading to an overuse of food supplements with CBD without solid evidence [21]. Although several narrative reviews have discussed the potential therapeutic applications of cannabinoids in oral health, a systematic synthesis specifically addressing the role of cannabidiol in periodontal disease remains limited [18,22,23]. The present review integrates recent experimental and clinical evidence on the antimicrobial, anti-inflammatory, and bone-protective effects of CBD within the pathophysiological framework of periodontitis. By analyzing data from cellular models, animal studies, and emerging clinical evidence, this review provides a comprehensive perspective on the potential of CBD as a host-modulatory and antibiofilm agent in periodontal therapy. Given the numerous products with cannabidiol commercialized as food supplements recommended as adjunctive in periodontitis, the objective of this systematic review is to summarize the state of the art regarding the implications of CBD in the treatment of periodontitis.

## 2. Methods

This systematic review was conducted according to PRISMA 2020 guidelines (PRISMA checklist is provided as Supplementary file). The review protocol was registered in the PROSPERO database (registration number: CRD420261330976). A comprehensive electronic search was conducted in Web of Science, Cochrane, PubMed, Scopus, and Google Scholar from 2006 to April 2026. The search strategy combined controlled vocabulary terms and free-text keywords related to cannabidiol and periodontal disease. Boolean operators (“AND”, “OR”) were used to combine search terms. The literature search was performed between 8 March 2026 and 30 April 2026. All retrieved records were imported into Mendeley reference management software, version 1.19.8 (Elsevier, Amsterdam, The Netherlands), for organization, and duplicate records were identified both automatically and manually and removed prior to title and abstract screening. Study selection was performed independently by two reviewers. Furthermore, a manual screening of the reference lists of included articles and relevant reviews was conducted to identify additional studies that may not have been retrieved through the electronic search strategy.

### 2.1. Eligibility Criteria

Studies were included if they: (i) investigated cannabidiol as a primary intervention; (ii) evaluated the levels of pro-inflammatory cytokines, microbial biofilm, or alveolar bone remodeling; (iii) were original research articles published in English.

Studies were excluded if they: (i) investigated other phytocannabinoids without CBD or evaluated whole *Cannabis* extract without specifying CBD content; (ii) focused solely on recreational *Cannabis* consumption without therapeutic evaluation; (iii) did not report quantitative or interpretable biological outcomes; (iv) were reviews, editorials, conference abstracts without full text, or case reports.

### 2.2. Study Selection and Data Extraction

All records retrieved from electronic databases were exported into the reference management software, and duplicates were removed. Full texts of potentially relevant studies were subsequently assessed against inclusion and exclusion criteria. Disagreements between reviewers were resolved through discussion or consultation with a third reviewer. The following information was collected: study design, experimental model, cannabidiol concentration and formulation, measured outcomes, and main findings. Methodological quality and risk of bias were assessed according to study design. Randomized clinical studies were evaluated using the Cochrane Risk of Bias Tool 2, while animal studies were assessed using the SYRCLE Risk of Bias Tool. Two reviewers independently performed study selection, data extraction, and methodological quality assessment. Discrepancies were resolved by consensus.

Given the anticipated heterogeneity in study designs, experimental models, and outcome measures, findings were synthesized narratively. Results were grouped according to anti-inflammatory and bone remodeling effects, antimicrobial and antibiofilm activity, and clinical periodontal data.

## 3. Results

### 3.1. Study Selection

**Figure 2:**
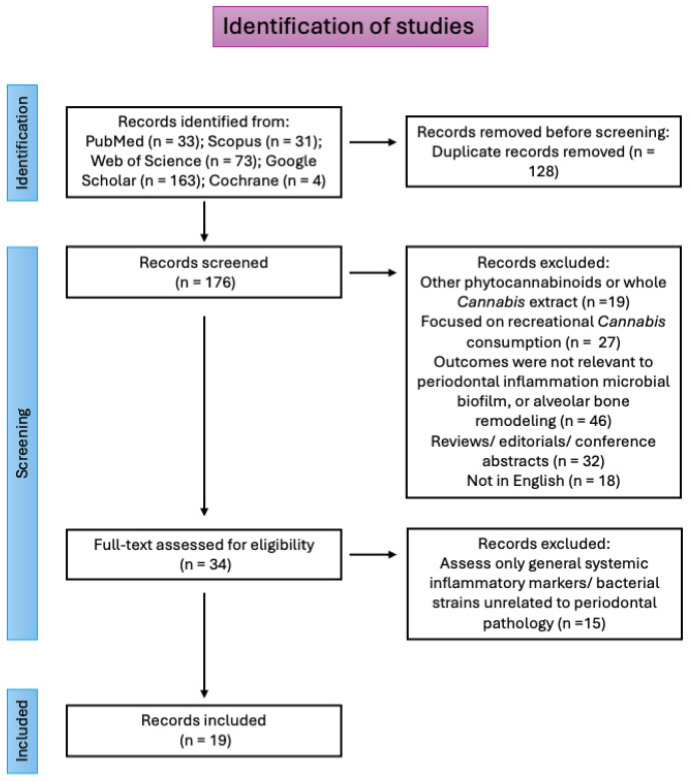
Flowchart of the selection process of studies.

The search identified 304 records, with 128 duplicates removed. After screening 176 records, 142 studies were excluded due to a lack of relevance or absence of CBD as the primary intervention, as can be seen in Figure 2. Thirty-four studies met the inclusion criteria and were included in the further step of full-text evaluation for eligibility, and from them, 19 studies were included in the qualitative analysis. The included studies comprised in vitro cellular models, animal models of periodontitis, and limited clinical investigations.

### 3.2. Characteristics of Included Studies

The included studies investigated the biological effects of cannabidiol using various experimental models, including human gingival fibroblasts, periodontal ligament cells, stem cells derived from periodontal tissues, oral biofilm systems, and animal models of experimental periodontitis (Table 1). Most studies focused on the molecular and cellular mechanisms through which CBD may influence periodontal inflammation, microbial biofilms, and bone remodeling processes.

In vitro studies mainly evaluated the effects of CBD on inflammatory cytokine production, cell proliferation, migration, and differentiation of periodontal-related cells. Several studies also investigated the antimicrobial activity of CBD against oral bacteria and its effects on multispecies biofilm models.

Animal studies examined the therapeutic potential of CBD in experimental periodontitis models, where CBD administration was associated with reduced inflammatory responses and decreased alveolar bone loss.

Only a limited number of clinical studies were identified; however, the available data suggest that topical cannabidiol formulations may improve periodontal clinical parameters, including gingival and bleeding indices. Two registered clinical trials (ClinicalTrials.gov IDs: NCT05498012 and NCT05646459) out of three identified had no results published to date, and one of them was withdrawn, highlighting the current gap between preclinical evidence and clinical validation [24,25].

**Table 1:** Characteristics of the included studies.

Study Type	Model	Dose	Route/Exposure	Outcome	Author (Year)
Animal	Ligature-induced rat periodontitis	CBD 5 mg/kg	Intra-periotoneal	CBD significantly inhibited the volume of bone loss; ↓RANKL, ↓RANK, ↓MPO activity, ↓TNF-α, ↓IL-1β	Napimoga, 2009 [26]
In vitro	Human gingival fibroblasts	CBD 0.05, 0.5, 1, 2 µM	Direct exposure	Modulated extracellular matrix metabolism (↑TGF-β, ↑fibronectin, altered MMP activity)	Rawal, 2012 [27]
In vitro	Human periodontal ligament mesenchymal stem cells (hPDLSCs)	CBD combined with moringin (48 h treatment)	Direct exposure	Increased stem cell survival, reduced apoptosis, and enhanced neuronal differentiation markers (Nestin, GAP43)	Cariccio, 2018 [28]
In vitro	IL-1β-stimulated human gingival fibroblasts	0.1–1 μg/mL CBD	Direct exposure	↓IFN-γ, ↓TNF-α, ↓IL-2; modulation of PGE2 production	Abidi, 2022 [29]
In vitro	Human gingival fibroblasts	~1 µg/mL	Direct exposure	Reduced pro-inflammatory cytokines (IFN-γ, TNF-α, IL-2) and modulated prostaglandin E2 production in IL-1β-stimulated fibroblasts	Compton, 2022 [30]
In vitro	LPS-stimulated RAW 264.7 macrophages + human gingival fibroblasts (HGF-1)	~0.5 µg/mL CBD	Direct exposure	Reduced TNF-α and IL-1β production and enhanced gingival fibroblast wound healing activity	Kongkadee et al., 2022 [31]
In vitro	Human gingival fibroblasts	CBD 0.01–30 µM	Direct exposure	Increased fibroblast proliferation and migration	Montreekachon, 2023 [32]
Animal	Experimental rat periodontitis/hPDLCs	CBD 5 mg/kg	Topical	Reduced bone loss and inflammation via inhibition of TLR4/NF-κB signaling	Chen, 2023 [33]
In vitro	Primary human osteoblasts are isolated from the alveolar bone	CBD 0.01–10 μM	Direct exposure	Increased osteoblast proliferation, enhanced osteogenic differentiation, and promoted biomineralization	Thanai-nopparat, 2023 [34]
In vitro	Human dental pulp stem cells (DPSCs)	CBD 0.1–12.5 µM	Direct exposure	Increased proliferation and migration, enhanced osteogenic/odontogenic differentiation, and reduced TNF-α-induced inflammatory cytokines (TNF-α, IL-1β, IL-6)	Yu, 2023 [35]
In vitro	Human gingival fibroblasts	CBD 0.25–0.5 µM	Direct exposure	Non-significant decrease in the production of IL-6 and IL-8, increase in the HMOX1 mRNA levels	Jirasek, 2024 [36]
Clinical	Patients with stage I-IV periodontitis	1% CBD dental gel and 1% CBD toothpaste	Topical	CBD showed anti-inflammatory effects in the gingival tissues	
In vitro	Human gingival fibroblasts and oral keratinocytes	CBD 1, 25, 50, 100 μM	Direct exposure	Dose-dependent effects: high concentrations (≥50 μM) induced apoptosis and DNA damage, while low concentrations (1 μM) were biocompatible	Pagano, 2024 [37]
In vitro	*Treponema denticola*	0.1–10 µg/mL CBD	Direct exposure	CBD induced differential expression of 392 genes related to stress response and toxin regulation; *T. denticola* showed resistance to CBD	Tan et al., 2024 [38]
In vitro	Periodontal ligament fibroblasts + multispecies oral biofilm	CBD 10–20 μM; 125–500 μg/mL	Direct exposure	Inhibited *S. mutans*, reduced biofilm metabolic activity, and modulated cytokine production	Garzon, 2024 [39]
In vitro	Human dental pulp stem cells (hDPSCs) stimulated with LPS	1.25, 2.5, 5, 10, 25, and 50 μg/mL CBD	Direct exposure	CBD restored proliferation, migration, and odonto/osteogenic differentiation inhibited by LPS and promoted mineralization markers	Kornsuthisopon, 2025 [40]
In vitro	Immortalized murine dental pulp cells + macrophages under inflammatory conditions	CBD 0.01–10 µM	Direct exposure	CBD enhanced biomineralization and modulated inflammatory mediator expression under pro-inflammatory conditions	Sales et al., 2025 [41]
In vitro	RAW 264.7 cells	CBD 10 µM + Taurine 0.5 mM CBD + taurine	Direct exposure	↓TNF-α, ↓IL-1β, TRAP + cells	Kim, 2025 [42]
Animal	*P. gingivalis*-induced rat periodontitis/RAW 264.7 cells	CBD 2 mg/kg + taurine 100 mg/kg CBD 20 mg/kg + taurine 100 mg/kg	Oral administration	Reduced TNF-α, IL-1β, TRAP+ cells and alveolar bone loss	
In vitro	Human bone stromal cells (jaw tori)	≤10 µM CBD (non-cytotoxic range) for 24–48 h	Direct exposure	Increased osteogenic differentiation and mineralization via AKT/β-catenin signaling, with upregulation of RUNX2, BSP, and Osterix.	Makeudom, 2026 [43]
In vitro	RAW 264.7/*Staphylococcus aureus*/*Porphyromonas gingivalis*	CBD-loaded bioinspired mucoadhesive nanomicelles	Direct exposure	Inhibited growth of periodontal pathogens and modulated inflammatory signaling	Liu, 2026 [44]
Animal	Mouse periodontitis model		Local periodontal injection/topical application	Reduced inflammation, promoted bone regeneration, and ↓ROS production	

### 3.3. Anti-Inflammatory and Bone Remodeling Effects

An important number of the included studies investigated the anti-inflammatory properties of cannabidiol in periodontal cells and tissues. Experimental findings demonstrated that CBD modulates inflammatory signaling pathways involved in periodontal disease. In vitro experiments using gingival fibroblasts showed that cannabidiol alters extracellular matrix metabolism and reduces matrix metalloproteinase activity, suggesting a potential role in limiting connective tissue degradation [30,31,32,36,37]. There are, however, contradictory results: for example, Rawal et al. have evaluated in vitro the effects of CBD on human gingival fibroblast and matrix-degrading enzymes, and have concluded that CBD increases TGFβ, MMP1, and MMP2 levels at low doses. Also, the authors reported an increase in fibronectin production, and their study suggests that CBD could promote fibrosis [27]. Interestingly, at higher doses, the effects were opposite. As can be seen in Table 1, multiple studies demonstrated that CBD reduces inflammatory mediator production in periodontal cells. Cannabidiol inhibited LPS-induced cytokine production and suppressed NF-κB activation, indicating a potential host-modulatory effect. In in vitro studies, CBD was typically applied directly to cultured cells or biofilm models at concentrations ranging approximately from 0.01 μM to 30 μM in human gingival fibroblast models and up to 125–500 μg/mL in multispecies oral biofilm systems. Lower micromolar concentrations (approximately 3–10 μM) were frequently reported to exert anti-inflammatory or proliferative effects in gingival fibroblasts without cytotoxicity.

Animal studies further supported the in vitro findings. Cannabidiol administration in experimental periodontitis models resulted in the reduced expression of pro-inflammatory cytokines and suppression of the RANK/RANKL pathway, a key regulator of osteoclast differentiation and bone resorption. Additionally, inhibition of the TLR4/NF-κB pathway was identified as a potential mechanism underlying the anti-inflammatory effects of cannabidiol in periodontal tissues [26,33,42,44]. Napimoga et al. have evaluated the effects of CBD on ligature-periodontal disease in rats, compared with a placebo. Their study has noticed that in the group treated with i.p. 5 mg CBD/kg daily, for 30 days, the animals had a significantly decreased alveolar bone loss, and the expression of the receptor activator of the nuclear factor-κB ligand and the decoy receptor RANKL/RANK was reduced. Also, the levels of pro-inflammatory cytokines IL-1 and TNF were lower in the CBD-treated group compared with the placebo. The authors also noticed a down-regulation of neutrophil migration, which can be the effect of the reduction in cytokine levels [26]. These results are in correlation with the results published by Ossola et al., which have evaluated the effects of a synthetic cannabinoid (methanandamide) in an induced model of periodontitis in rats [45]. Daily application of methanandamide leads to decreased alveolar bone loss compared with the placebo. The authors also reported that the treatment was able to decrease the expression of TNF-alpha, PGE_2_, and NO [45]. Stimulation of cannabinoid receptors CB1 and CB2 determines a reduction in the synthesis of pro-inflammatory mediators [7]. Also, the activation of cannabinoid receptor CB2 in periodontal ligament cells promotes osteogenic differentiation and increases the OPG/RANKL ratio, creating a microenvironment favorable for bone formation [46]. Together, these findings indicate that cannabidiol may act as a host-modulatory agent, attenuating inflammatory signaling pathways associated with periodontal tissue destruction and also highlight the potential role of cannabinoid systems in regulating alveolar bone metabolism in periodontal tissue.

Although cited as a human study, the research conducted by Vasudevan and Stahl included humans, but only for sampling of the dental plaque for culturable bacteria [47].

### 3.4. Antibacterial Activity

Several studies evaluated the antimicrobial activity of cannabidiol against oral microorganisms, including key periodontal pathogens.

In the research where extracts from *Cannabis* sp. have been tested, the results could be biased by the other compounds found in the extracts. It is worth noting that extracts could contain volatile compounds from the essential oil, compounds that are powerful antimicrobial agents. The main compounds found in the essential oil are myrcene, alpha-pinene, and beta-pinene, and these compounds have strong antibacterial activity [48,49]. The main mechanism of action by which these terpenes inhibit bacterial growth is the membrane destruction.

Blaskovich et al. have evaluated the antimicrobial potential of CBD [50]. Their complex study has emphasized that CBD has potent antibacterial activity against many Gram-positive bacteria, but also against four Gram-negative strains. The study concluded that the topical use could be beneficial in different skin infections. Extrapolating the results, we can assume that CBD could be successfully used in periodontal disease [50].

Moreover, as Kosgodage et al. have shown, CBD can be used as a bacterial sensitizer to antibiotics, via the modulation of membrane vesicle release [51].

The pathogenic bacteria implicated in the pathophysiology of periodontitis are mainly anaerobic Gram-negative: *Actinobacillus actinomycetemcomitans*, *Bacteroides forsythus*, *Eikenella corrodens*, *Fusobacterium nucleatum*, *Porphyromonas gingivalis*, *Prevotella intermedia,* and *Treponema denticola* [5,52]. Specific studies targeting periodontal pathogens confirmed that cannabidiol can inhibit biofilm formation by periodontopathogenic species and modulate bacterial gene expression. Transcriptomic analyses revealed alterations in bacterial stress response pathways following exposure to cannabidiol, suggesting that cannabinoids may disrupt bacterial homeostasis. In addition, investigations using dental plaque samples showed that cannabinoid-containing mouthwash formulations significantly reduced culturable bacterial load, with efficacy comparable to chlorhexidine in some experimental settings. According to Tolentino et al., CBD can reduce the abundance of key periodontal pathogens in polymicrobial biofilms. The study tested the antimicrobial activity of Cannabidiol in a multispecies subgingival biofilm model containing 33 bacterial species. The results showed that CBD significantly reduced the total bacterial count and decreased the proportion of the “red complex” bacteria, which are strongly associated with periodontal disease. Specifically, CBD reduced important periodontal pathogens such as *Porphyromonas gingivalis* and *Tannerella forsythia*, suggesting that CBD may interfere with the microbial dysbiosis involved in periodontitis [53].

## 4. Discussion

**Figure 3:**
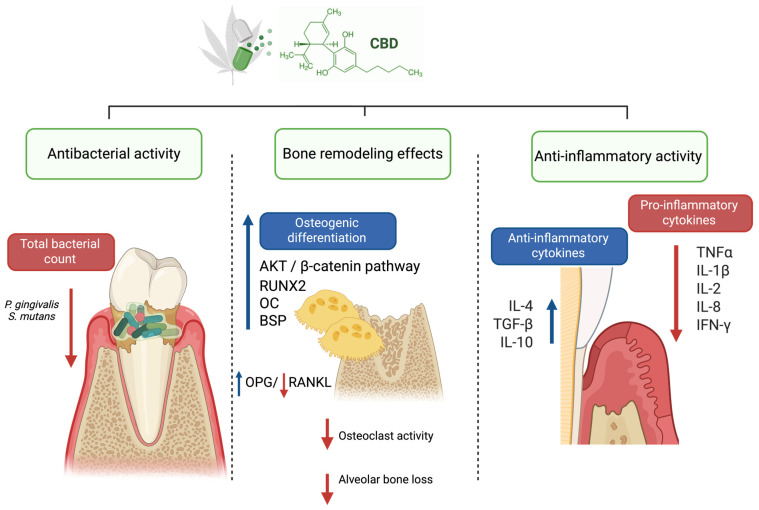
Modulatory effects of CBD in periodontal disease. Created in BioRender. Stefanescu, R. (2026), https://BioRender.com/np2zhx5 (accessed on 12 March 2026).

The present systematic review provides a comprehensive overview of the current evidence regarding the biological effects of cannabidiol in periodontal-related models. The main findings indicate that CBD exhibits anti-inflammatory, antimicrobial, and bone-modulating properties that may be relevant to the pathophysiology of periodontal disease (Figure 3).

Although the included studies generally support the anti-inflammatory potential of cannabidiol, the consistency of the reported findings varied depending on the evaluated cytokine, experimental model, and treatment conditions. Among the investigated inflammatory mediators, inhibition of TNF-α and IL-1β was one of the most consistently observed effects across both in vitro and animal models of periodontal inflammation [26,29,30,33,35,42]. Since these cytokines play central roles in periodontal tissue destruction and osteoclastogenic signaling, their modulation may represent one of the principal mechanisms underlying the protective effects of CBD in periodontal tissues.

In contrast, the effects of cannabidiol on IL-6 appeared less consistent. While several studies demonstrated reduced IL-6 production following CBD exposure [36], other investigations reported minimal changes or even increased IL-6 expression under specific experimental conditions [30]. This variability may reflect differences in cell type, inflammatory stimulus, cannabidiol concentration, exposure duration, or the dual biological role of IL-6 itself, which may exert both pro-inflammatory and immunoregulatory functions depending on the context.

Similar variability was observed regarding the reported effects of cannabidiol on osteoclastogenesis and bone remodeling. In vitro studies consistently demonstrated enhanced osteogenic differentiation and biomineralization, including the upregulation of markers such as RUNX2, osteocalcin, and bone sialoprotein [35,43]. These findings were partially supported by animal studies demonstrating reduced alveolar bone loss and decreased osteoclast activity in experimental periodontitis models [26,33,42].

An important consideration is the presence of contradictory or unresolved findings within the literature. While many studies reported anti-inflammatory, regenerative, and osteogenic effects of CBD, others demonstrated dose-dependent cytotoxicity, apoptosis induction, or potential fibrosis-related responses in gingival fibroblasts. For example, Rawal et al. observed increased transforming growth factor-β and fibronectin production together with altered matrix metalloproteinase activity following CBD exposure, suggesting a potential profibrotic effect in gingival fibroblasts [27].

These observations indicate that the biological effects of cannabidiol in periodontal-related tissues are likely context-dependent. Therefore, although the available evidence suggests promising anti-inflammatory and bone-modulating properties, the current literature remains predominantly preclinical and methodologically heterogeneous. Consequently, caution should be exercised to avoid overgeneralization of the therapeutic potential of cannabidiol in periodontal therapy until further standardized and clinically relevant studies become available.

In addition to its anti-inflammatory properties, CBD has also demonstrated antimicrobial and antibiofilm activity against oral microorganisms. Some studies showed that CBD inhibited bacterial growth and reduced the metabolic activity of multispecies oral biofilms [42,44]. As expected, the antibacterial activity appears to be species dependent. For example, transcriptomic studies have shown that certain periodontal pathogens, including *Treponema denticola*, may exhibit resistance to CBD while activating stress–response pathways, indicating that the antimicrobial activity of cannabinoids may not be universal across all periodontal pathogens [38].

Nevertheless, some studies reported dose-dependent or opposing cellular responses to CBD exposure. For example, while low concentrations of CBD were generally well tolerated and sometimes promoted cell proliferation or migration, higher concentrations were associated with cytotoxic effects, apoptosis induction, and DNA damage in gingival fibroblasts [37]. Similarly, certain studies suggested that CBD may stimulate extracellular matrix production and fibrotic responses in gingival fibroblasts, indicating that its biological effects may vary depending on cell type, concentration, and experimental conditions.

These discrepancies highlight the complexity of cannabinoid signaling in oral tissues and suggest that the therapeutic effects of CBD may depend strongly on dose, delivery route, and local microenvironmental conditions. Further research is therefore needed to determine the optimal therapeutic concentrations and to better understand the molecular pathways involved.

Although CBD has been investigated in numerous clinical contexts, evidence specifically addressing its effects in periodontitis remains scarce. The clinical study conducted in 2022 (ClinicalTrials.gov Identifier: NCT05498012) that aimed to evaluate the effects of CBD in periodontitis has no published results yet, and another clinical study with a similar topic was withdrawn (ClinicalTrials.gov Identifier: NCT05646459) due to legal aspects. The limited available clinical evidence, such as the study conducted by Jirasek et al., suggests potential improvements in periodontal clinical parameters following topical CBD application, although these findings remain preliminary, and underline the lack of validated clinical evidence in periodontal therapy.

The route of cannabidiol administration varied considerably across the included animal studies, which may contribute to differences in therapeutic outcomes. Among the four identified studies, cannabidiol was delivered via local periodontal injection or topical application in two studies, orally in one study, and intraperitoneally in another. This heterogeneity highlights the absence of standardized delivery protocols and complicates a direct comparison between studies. Importantly, given the localized nature of periodontal disease, locally applied formulations may offer advantages by achieving higher concentrations at the site of inflammation while minimizing systemic exposure. In contrast, systemic administration routes, such as oral or intraperitoneal delivery, may result in lower bioavailability at the periodontal level and increased variability due to metabolic factors [54,55,56]. In this context, local delivery systems such as cannabinoid-containing gels, mouthwashes, biodegradable fibers, or nanoparticle-based carriers may provide sustained release of CBD within periodontal pockets and enhance therapeutic efficacy [57,58].

Several challenges related to its local administration and clinical translation in periodontal therapy remain insufficiently addressed. Cannabidiol is a highly lipophilic compound with limited aqueous solubility, which may restrict its diffusion and retention within the periodontal microenvironment. In addition, salivary flow, moisture, pH fluctuations, enzymatic activity, and microbial biofilms may further affect CBD stability, tissue penetration, and sustained therapeutic activity [59,60].

These limitations highlight the need for optimized local delivery systems, such as hydrogels, nanoparticles, nanomicelles, and bioadhesive carriers, which may improve CBD bioavailability, stability, and controlled release within periodontal tissues [61,62]. However, the pharmacokinetic behavior of CBD in periodontal tissues remains poorly characterized, and limited information is available regarding tissue penetration, long-term stability, and local drug distribution. Additional translational challenges include the lack of standardized clinical protocols, variability in formulation composition and manufacturing quality, and the limited regulatory approval of CBD-containing periodontal products. Although most studies reported acceptable short-term biocompatibility at lower concentrations, long-term safety data remain scarce, and some investigations demonstrated dose-dependent cytotoxic or fibrosis-related effects under specific experimental conditions.

Taken together, the current evidence suggests that cannabidiol may influence several biological processes involved in periodontal disease, including inflammatory signaling, microbial biofilm dynamics, and bone remodeling. However, further well-designed preclinical and clinical studies are required to clarify its therapeutic potential and to establish safe and effective protocols for periodontal applications.

### 4.1. Limitations of the Current Evidence

Several limitations should be considered when interpreting the findings of this review. Firstly, a significant proportion of the included studies were in vitro or preclinical animal studies, which may not fully replicate the complex microbial and immunological environment of human periodontal disease. Secondly, the heterogeneity in experimental designs, outcome measures, and cannabidiol concentrations prevented quantitative synthesis of the results. Considerable variability was observed regarding the experimental models employed, including gingival fibroblasts, periodontal ligament cells, stem cells, multispecies biofilm systems, and animal models of experimental periodontitis. In addition, CBD concentrations differed markedly across studies, ranging from low micromolar concentrations in cellular experiments to systemic administration in animal models. This methodological heterogeneity complicates direct comparison between studies and limits the ability to draw definitive conclusions regarding optimal dosing strategies, administration routes, and therapeutic efficacy. The variability in experimental conditions may partially explain the occasionally contradictory findings. Therefore, the current evidence should be interpreted with caution, and future investigations should aim to establish more standardized experimental protocols.

Finally, the limited number of clinical trials restricts the ability to draw clear conclusions regarding the therapeutic efficacy of cannabidiol in periodontal therapy.

### 4.2. Challenges and Future Perspectives

CBD local administration can be a potential adjunctive strategy in the management of periodontitis. As future perspectives, clinical studies should focus on randomized clinical trials, on optimized delivery systems, and dose-dependent relationship studies. Although not completely understood, the mechanisms of action discovered in the in vitro studies and in the preclinical studies provide sufficient proof for this compound to be further investigated in clinical studies. To address whether cannabidiol represents a viable therapeutic option for periodontitis or whether its perceived benefits are influenced by current trends, it is important to acknowledge that, although interest in CBD and related products may at times be driven by overuse or bias, the available scientific evidence suggests a genuine potential for its development as a novel therapeutic approach in periodontal therapy.

## 5. Conclusions

Cannabidiol is a multi-target phytotherapeutic agent with anti-inflammatory, antibacterial, and host-modulatory properties. Experimental studies indicate that CBD may attenuate inflammatory signaling pathways, inhibit biofilm formation, and reduce alveolar bone resorption, suggesting potential relevance for periodontal therapy. In addition, CBD has been reported to accumulate relatively high concentrations within the oral mucosa, supporting its potential suitability for local periodontal applications.

Nevertheless, the currently available evidence remains predominantly preclinical and provides mainly mechanistic insights derived from in vitro and animal models. Consequently, several proposed applications, including CBD-containing hydrogels, nanoparticles, and local periodontal delivery systems, should presently be regarded as exploratory rather than clinically validated approaches. Although regenerative, anti-inflammatory, and osteogenic effects have been demonstrated experimentally, further well-designed clinical studies are required before cannabidiol can be recommended as a therapeutic agent in routine periodontal practice.

## Data Availability

No new data were created or analyzed in this study.

## Supplementary Materials

The following supporting information can be downloaded at https://www.mdpi.com/article/10.3390/biomedicines14051163/s1. PRISMA checklist.

## Abbreviations

The following abbreviations are used in the manuscript:

AKT	Protein kinase B
BSP	Bone sialoprotein
CB1	Cannabinoid receptor 1
CB2	Cannabinoid receptor 2
CBD	Cannabidiol
GAP43	Growth-associated protein 43
hDPSCs	Human dental pulp stem cells
HMOX-1	Heme oxygenase-1
hPDLCs	Human periodontal ligament cells
IFN-γ	Interferon-gamma
IL-1β	Interleukin-1 beta
IL-2	Interleukin-2
IL-6	Interleukin-6
LPS	Lipopolysaccharide
MMP	Matrix metalloproteinase
MPO	Myeloperoxidase
NF-κB	Nuclear factor-κB
NO	Nitric oxide
OC	Osteoclacin
OPG	Osteoprotegerin
PGE2	Prostaglandin E2
RANK	Receptor activator of nuclear factor-κB
RANKL	Receptor activator of NF-κB ligand
RUNX2	Runt-related transcription factor 2
TGF-β	Transforming growth factor-beta
TLR4	Toll-like receptor 4
TNF-α	Tumor necrosis factor-alpha
TRAP+	Tartrate-resistant acid phosphatase positive
